# Mortality from and Incidence of Pesticide Poisoning in South Korea: Findings from National Death and Health Utilization Data between 2006 and 2010

**DOI:** 10.1371/journal.pone.0095299

**Published:** 2014-04-17

**Authors:** Eun Shil Cha, Young-Ho Khang, Won Jin Lee

**Affiliations:** 1 Department of Preventive Medicine, College of Medicine, Korea University, Seoul, South Korea; 2 Department of Preventive Medicine, University of Ulsan College of Medicine, Seoul, South Korea; 3 Institute of Health Policy and Management, Seoul National University College of Medicine, Seoul, South Korea; Centers for Disease Control and Prevention, United States of America

## Abstract

Pesticide poisoning has been recognized as an important public health issue around the world. The objectives of this study were to report nationally representative figures on mortality from and the incidence of pesticide poisoning in South Korea and to describe their epidemiologic characteristics. We calculated the age-standardized rates of mortality from and the incidence of pesticide poisoning in South Korea by gender and region from 2006 through 2010 using registered death data obtained from Statistics Korea and national healthcare utilization data obtained from the National Health Insurance Review and Assessment Service of South Korea. During the study period of 2006 through 2010, a total of 16,161 deaths and 45,291 patients related to pesticide poisoning were identified, marking respective mortality and incidence rates of 5.35 and 15.37 per 100,000 population. Intentional self-poisoning was identified as the major cause of death due to pesticides (85.9%) and accounted for 20.8% of all recorded suicides. The rates of mortality due to and incidence of pesticide poisoning were higher in rural than in urban areas, and this rural-urban discrepancy was more pronounced for mortality than for incidence. Both the rate of mortality due to pesticide poisoning and its incidence rate increased with age and were higher among men than women. This study provides the magnitude and epidemiologic characteristics for mortality from and the incidence of pesticide poisoning at the national level, and strongly suggests the need for further efforts to prevent pesticide self-poisonings, especially in rural areas in South Korea.

## Introduction

Pesticide poisoning has been recognized as an important public health issue around the world [1]. Approximately 350,000–440,000 annual suicides by means of deliberate pesticide poisoning have been estimated to occur worldwide [2], and the numbers of victims of nonfatal pesticide poisoning are assumed to be much greater. However, few studies [3]–[5] have been conducted to evaluate nationally representative figures of acute pesticide poisoning. Identifying at the national level the magnitude of fatal and nonfatal pesticide poisoning and high risk individuals would well provide scientific evidence regarding the overall disease burden and serve as a fundamental step forward in developing national strategies to curb this grave issue.

Previously, we reported the total number of pesticide poisoning deaths from 1996 through 2005 to be 25,360 and that the age-standardized mortality rates due to pesticide poisoning significantly increased during this period from 4.42 to 6.42 per 100,000 population, accounting for the largest proportion of deaths among all forms of poisoning [6]. We also estimated an annual rate of pesticide-related hospitalization in South Korea of 17.8 per 100,000 population [7] and of the emergency department visit of 26.8 per 100,000 population [8]. However, these figures were derived from sampled hospitals accounting for about 30% of all hospitals across South Korea. Moreover, relatively recent figures on the time trends in mortality from pesticide poisoning in South Korea have not been presented since 2005.

Therefore, we aimed to update the rate of mortality using registered death data and investigate the incidence and describe the epidemiological characteristics of pesticide poisoning using National Health Insurance claims data, which cover the entire population of South Korea.

## Methods

### Mortality Data

By law, all deaths of South Koreans must be reported to Statistics Korea within one month of their occurrence. Mortality data for South Korea between 2006 and 2010 were obtained from the registered death data provided by Statistics Korea [9]. The registered death data include information on age, gender, administrative district of residence, educational level, month of death, occupation, and marital status. Underlying causes of disease are coded in the data according to the *International Classification of Diseases and Related Health Problems, 10th Revision* (ICD-10) [10]. Pesticide poisoning deaths were defined as those featuring the code for toxic effect of pesticides (T60.0–T60.9). External cause of injury codes were used to classify the causes of pesticide poisoning deaths into intentional self-poisoning (X68) and unintentional poisoning [i.e., accidental poisoning (X48), assault (X87), and undetermined intent poisoning by and exposure to pesticides (Y18)]. Based on the 251 administrative residential districts used in the death data, urbanity levels were identified. Metropolises included Seoul and six other metropolitan cities in South Korea. Districts in the nine provinces other than these metropolises were classified into either cities or rural areas depending on governmental administrative divisions determined by population size and rural characteristics.

### National Health Insurance claims data

The National Health Insurance of South Korea was established in 1989 and by statute covers the entire South Korean population and all medical institutions in the nation. Health care utilization data were extracted from the Korea National Health Insurance claims database retrieved from the National Health Insurance Review and Assessment Service between 2006 and 2010. All inpatient and outpatient medical utilization related to pesticide poisoning is expected to be included in the database. The National Health Insurance claims data contain primary and additional diagnostic codes per ICD-10. Identical ICD-10 codes (T60.0–T60.9) as in mortality data were used for this incidence data. The initial episode of emergency department visit or hospital admission during the study period of January 1, 2006 through December 31, 2010 was treated as one incident case of pesticide poisoning when a patient was examined at multiple hospitals or at a single hospital for several different conditions. In National Health Insurance claims data, only few records were obtained by external cause of injury code, and it was nearly impossible to differentiate intention of pesticide poisoning. The National Health Insurance claims data also contained information on types of care received, dates of hospitalization, address of medical institutions, and patients’ genders and ages. Due to the lack of information regarding patients’ addresses in our data, the locations of medical institutions were used as a proxy measure for patients’ residential area and classified into metropolis, small/medium city, and rural area. The season in which pesticide poisoning occurred was extracted using the month of the occurrence of hospital visit.

### Data Analysis

The rates of mortality and incidence were directly standardized to 10-year age groups, using the 2000 World Standard Population [11] as the standard population in this direct standardization. The population data and the calculation process of age-standardized rate for pesticide poisoning were presented as Appendix (Tables S1 and S2 in Appendix S1). Population registration data by gender, 10-year age groups, and the 251 administrative districts were obtained from Statistics Korea [9]. The annual rates of pesticide poisoning mortality and incidence were calculated as number of deaths and incident cases per 100,000 population, and were stratified by gender and urbanization level. Descriptive statistics by sociodemographic variables for pesticide poisoning were presented. All statistical tests were performed via STATA, version 12.0 (StataCorp, College Station, Texas).

### Ethics Approval

We used publicly available mortality and healthcare utilization data without any personal identifiers, and thus ethical approval was unnecessary.

## Results

As shown in Table 1, a total of 16,161 deaths caused by pesticide poisoning were identified during the study period between 2006 and 2010, with an average annual death rate due to pesticide poisoning of 5.35 per 100,000 population. Those rates declined slightly from 5.74 to 4.85 per 100,000 population over the course of the five years. Intentional pesticide poisoning was the major cause of death from pesticide poisoning, accounting for 85.9% of all pesticide poisoning deaths (13,890 deaths over five years). The number of patients with pesticide poisoning found in the National Health Insurance data was 45,291 during the study period, with an average annual incidence rate of 15.37 per 100,000 population. The rates remained generally stable over the five years, with a slight decline witnessed in 2010.

**Table 1:** Age-standardized mortality and incidence rate of pesticide poisoning per 100,000 population by year in South Korea, 2006–2010.

	Mortality						Incidence					
	Total (T60)		Intentional (X68)		Unintentional (X48, X87, Y18)		Total		Hospitalization		Outpatient	
Year	Cases	ASR^a^	Cases	ASR	Cases	ASR	Cases	ASR	Cases	ASR	Cases	ASR
		(95% CI)		(95% CI)		(95% CI)		(95% CI)		(95% CI)		(95% CI)
2006	3,201	5.74	2,747	4.91	454	0.83	9,186	16.57	4,999	8.97	4,187	7.60
		(5.54–5.94)		(4.72–5.09)		(0.75–0.91)		(16.23–16.92)		(8.72–9.22)		(7.37–7.83)
2007	3,288	5.68	2,881	4.96	407	0.72	9,660	16.87	5,185	8.98	4,475	7.89
		(5.49–5.88)		(4.78–5.14)		(0.65–0.79)		(16.53–17.21)		(8.74–9.23)		(7.66–8.13)
2008	3,296	5.50	2,800	4.66	496	0.84	10,046	17.16	5,638	9.50	4,408	7.66
		(5.31–5.68)		(4.48–4.83)		(0.76–0.91)		(16.82–17.50)		(9.25–9.75)		(7.43–7.89)
2009	3,170	5.07	2,743	4.38	427	0.68	9,921	16.45	5,732	9.35	4,189	7.09
		(4.89–5.24)		(4.22–4.55)		(0.62–0.75)		(16.12–16.78)		(9.11–9.60)		(6.87–7.31)
2010	3,206	4.85	2,719	4.11	487	0.73	9,367	14.65	5,240	8.12	4,127	6.53
		(4.68–5.02)		(3.96–4.27)		(0.67–0.80)		(14.35–14.95)		(7.90–8.35)		(6.32–6.73)
Total	16,161	5.35	13,890	4.59	2,271	0.76	45,291^b^	15.37	26,274	8.81	19,016	6.56
		(5.27–5.44)		(4.52–4.67)		(0.73–0.79)		(15.22–15.51)		(8.70–8.91)		(6.47–6.66)

a Age-standardized rates per 100,000 population using the 2000 World Standard Population.

b Excluding duplicated patients during the study period, 2006–2010.

Table 2 illustrates that pesticide poisoning accounted for 66.9% of total poisoning deaths, whereas only 7.5% of all incident poisoning cases were due to pesticides. The proportion of death from pesticide poisoning among total poisoning deaths was similar between men and women (66.8% and 67.0%, respectively), but the proportion of incidence was higher among men than women (8.3% and 6.5%, respectively). The proportion of pesticide poisoning among total poisoning increased with age for both mortality and incidence data. Rural areas recorded the highest proportion (79.1%) of pesticide poisoning deaths among total poisoning deaths, but this was not the case with incidence. The proportion of pesticide poisoning among total suicide deaths was 20.8% overall and showed a wide variation between the 9.3% found in metropolises and 47.4% in rural areas.

**Table 2:** The proportion of pesticide poisoning among total poisoning, and total suicides by sex, age, and area of residence in South Korea, 2006–2010.

	Death cases				Incident cases	
	Pesticide poisoning deaths	PPP^a^	Suicide by pesticides	PPS^b^	Pesticide poisoning cases	PPP
	Number (%)	%	Number (%)	%	Number (%)	%
**Total**	16,161 (100)	66.9	13,890 (100)	20.8	45,291 (100)	7.5
**Sex**						
Men	10,844 (67.1)	66.8	9,361 (67.4)	21.5	26,855 (59.3)	8.3
Women	5,317 (32.9)	67.0	4,529 (32.6)	19.5	18,436 (40.7)	6.5
**Age group**						
0–9	7 (0.1)	11.9	0 (0.0)	0.0	1,256 (2.8)	2.0
10–19	40 (0.2)	21.4	35 (0.3)	2.1	510 (1.1)	1.4
20–29	282 (1.7)	26.3	265 (1.9)	3.4	1,702 (3.8)	3.0
30–39	1,033 (6.4)	44.4	935 (6.7)	8.8	4,318 (9.5)	5.4
40–49	2,211 (13.7)	57.7	2,015 (14.5)	16.0	8,254 (18.2)	7.3
50–59	2,564 (15.9)	66.9	2,290 (16.5)	21.0	8,449 (18.7)	8.0
60–69	3,570 (22.1)	75.3	3,076 (22.2)	31.9	9,500 (21.0)	11.6
70–79	4,235 (26.2)	80.5	3,536 (25.4)	40.0	8,077 (17.8)	15.6
≥80	2,218 (13.7)	77.6	1,737 (12.5)	36.7	3,224 (7.1)	21.4
**Area**						
Rural	5,488 (34.0)	79.1	4,560 (32.8)	47.4	11,137 (24.6)	7.1
City	7,808 (48.3)	68.6	6,812 (49.0)	22.8	24,915 (55.0)	8.6
Metropolis	2,865 (17.7)	49.3	2,518 (18.2)	9.3	9,239 (20.4)	5.8

a Proportion of pesticide poisoning among total poisoning.

b Proportion of pesticide poisoning among total suicide deaths.

**Figure 1:**
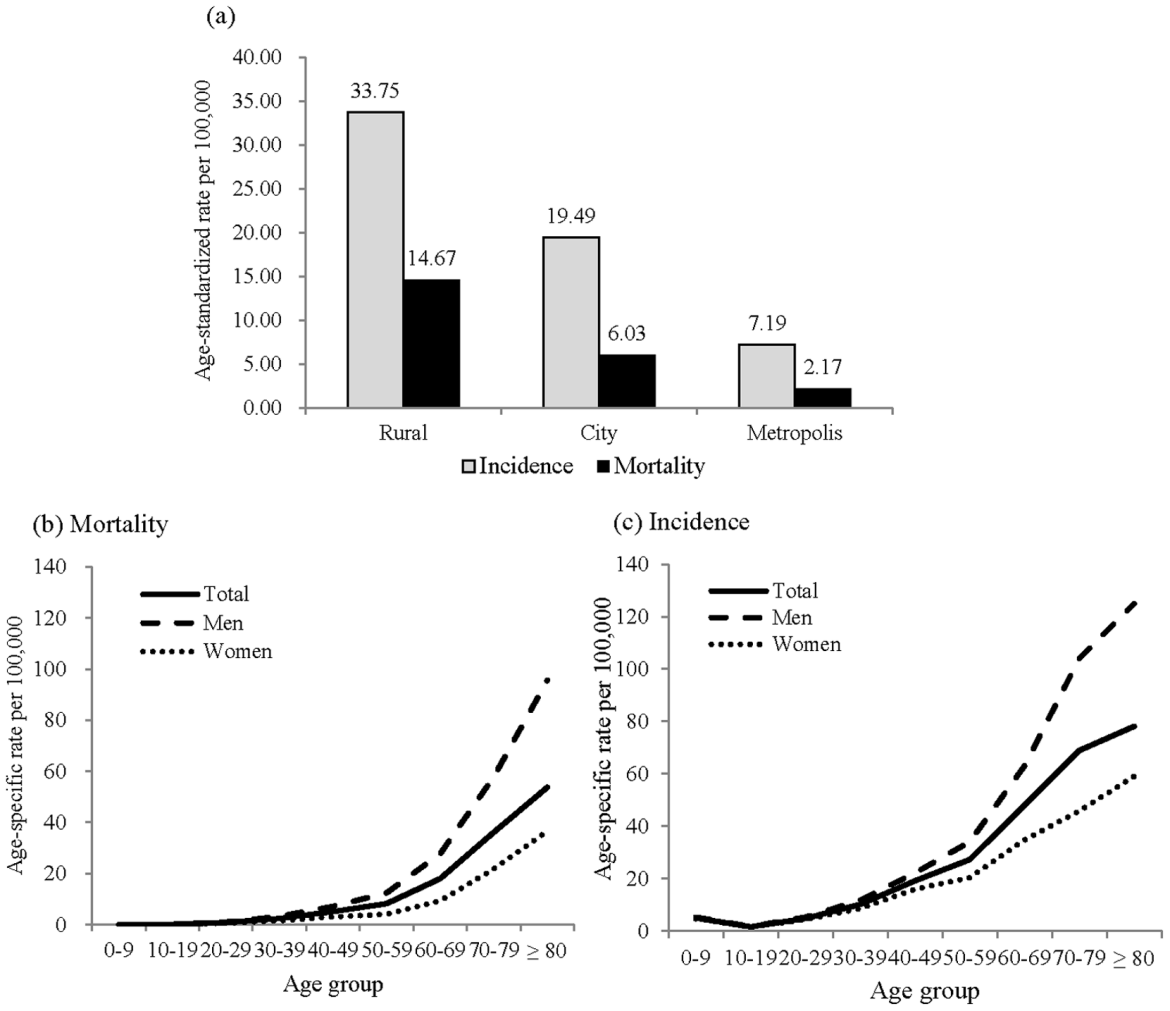
(a) Age-standardized incidence and mortality rate of pesticide poisoning per 100,000 population by area, (b) Age-specific mortality and (c) incidence rate of pesticide poisoning per 100,000 population by sex in South Korea, 2006–2010.

Figure 1 shows urban-rural distinctions in and age patterns of pesticide poisoning mortality and incidence. Both mortality and incidence showed the highest rates in rural areas, with 14.67 and 33.75 per 100,000 population, respectively, while those in metropolises showed the lowest (2.17 per 100,000 population for mortality and 7.19 per 100,000 population for incidence) (Figure 1 (a)). The rural-metropolis ratio in the rate of pesticide poisoning appears to be more pronounced in mortality (14.67/2.17 = 6.76) than in incidence (33.75/7.19 = 4.69). The mortality and incidence rates of pesticide poisoning exponentially increased with age and were higher among men than women across all age groups (Figure 1 (b), (c)). When we calculated the age-specific mortality rate by intention of death, the mortality rate for intentional self-poisoning demonstrated a greater increase with age compared to unintentional pesticide poisoning deaths (data not shown here).

As shown in Table 3, the mortality rate of intentional pesticide poisoning was the highest (11.25 per 100,000 population) in rural areas, followed by the rates in cities (5.25 per 100,000 population) and metropolises (1.95 per 100,000 population). Regardless of urbanization level, the mortality rate of intentional pesticide poisoning was higher among men than women, and the age-specific rate increased with age. In rural areas, the majority of pesticide poisonings occurred among agricultural workers and the unemployed, and herbicides and fungicides were most-widely used by suicide victims, but the roles of these agents were more important in mortality than in incidence (data not shown here). Similar seasonal variations were observed for all areas.

**Table 3:** Geographic difference of intentional pesticide poisoning in South Korea, 2006–2010.

Characteristic	Total	Rural	City	Metropolis
**Total**	13,890 (100)	4,560 (100)	6,812 (100)	2,518 (100)
**ASR (95% CI)** a				
Total	4.59	11.25	5.25	1.95
	(4.52–4.67)	(10.90–11.61)	(5.12–5.37)	(1.88–2.03)
Men	7.07	16.58	8.04	3.05
	(6.93–7.22)	(15.97–17.19)	(7.80–8.28)	(2.90–3.20)
Women	2.69	6.85	3.09	1.09
	(2.61–2.77)	(6.45–7.25)	(2.96–3.22)	(1.01–1.17)
**Age-specific rate** b				
0–19	0.06	0.4	0.05	0.01
20–29	0.7	3.1	0.9	0.3
30–39	2.2	9.9	2.5	0.9
40–49	4.7	18.9	5.0	2.1
50–59	7.4	21.4	8.9	3.3
60–69	15.5	37.1	18.1	6.5
70–79	30.2	54.9	35.2	12.1
≥80	42.1	85.5	45.3	15.4
**Occupation (case, %)**				
Managers	58 (0.4)	11 (0.2)	30 (0.4)	17 (0.7)
Professionals (including technicians)	210 (1.5)	40 (0.9)	113 (1.7)	57 (2.3)
Clerical support workers	325 (2.3)	59 (1.3)	180 (2.6)	86 (3.4)
Service and sales workers	668 (4.8)	166 (3.6)	318 (4.7)	184 (7.3)
Skilled agricultural, forestry and fishery workers	3,923 (28.2)	2,052 (45.0)	1,736 (25.5)	135 (5.4)
Craft and related trades workers	229 (1.7)	45 (1.0)	130 (1.9)	54 (2.1)
Plant and machine operators, and assemblers	106 (0.8)	13 (0.3)	68 (1.0)	25 (1.0)
Elementary occupations	519 (3.7)	153 (3.4)	267 (3.9)	99 (3.9)
Unemployed (including students and homemakers)	7,411 (53.4)	1,864 (40.9)	3,772 (55.4)	1,775 (70.5)
Unknown, Military	441 (3.2)	157 (3.4)	198 (2.9)	86 (3.4)
**Causative agents (case, %)**				
Organophosphate and carbamate insecticides (T60.0)	1,449 (10.4)	345 (7.6)	602 (8.8)	502 (19.9)
Halogenated insecticides (T60.1)	22 (0.1)	4 (0.1)	7 (0.1)	11 (0.5)
Other insecticides (T60.2)	112 (0.8)	31 (0.7)	57 (0.8)	24 (1.0)
Herbicides and fungicides (T60.3)	9,329 (67.2)	3,348 (73.4)	4,704 (69.1)	1,277 (50.7)
Rodenticides (T60.4)	25 (0.2)	9 (0.2)	10 (0.2)	6 (0.2)
Other pesticides (T60.8)	206 (1.5)	47 (1.0)	96 (1.4)	63 (2.5)
Pesticides, unspecified (T60.9)	2,747 (19.8)	776 (17.0)	1,336 (19.6)	635 (25.2)
**Season (case, %)**				
Spring	4,026 (29.0)	1,344 (29.5)	1,954 (28.7)	728 (28.9)
Summer	4,215 (30.4)	1,403 (30.8)	2,073 (30.4)	739 (29.4)
Fall	3,255 (23.4)	1,045 (22.9)	1,623 (23.8)	587 (23.3)
Winter	2,394 (17.2)	768 (16.8)	1,162 (17.1)	464 (18.4)

a Age-standardized rate per 100,000 population using the 2000 World Standard Population.

b Age-specific rate per 100,000 population.

## Discussion

The results of this study indicate that pesticide poisoning is a prevalent public health problem, resulting in 5.35 deaths per 100,000 population and 15.37 incidence cases per 100,000 population annually in South Korea during the period of 2006–2010. Pesticide poisoning accounted for a large portion of suicide in South Korea. Pesticide poisoning rates were higher in rural areas and among the elderly, agricultural workers, and unemployed individuals. This study presented pesticide poisoning mortality and incidence simultaneously at the national level and emphasized the urgency of constructing preventive national strategies to reduce pesticide poisoning in South Korea.

Although the mortality and incidence of pesticide poisoning declined during the study period, our estimates are much greater than the rates derived in a number of other developed countries such as Taiwan [3], Japan [4], and US [5], [12]. The magnitudes of pesticide poisoning mortality (5.35 per 100,000 population) shown in our study surpass those of a number of major causes of deaths in South Korea in 2010, such as breast cancer (mortality rate of 4.0 per 100,000 population), and Alzheimer’s disease (mortality rate of 4.8 per 100,000 population) [9].

The most important reason underlying this high mortality from pesticide poisoning is the elevated proportion of suicide by means of ingestion of pesticides, which accounted for 85.9% of total pesticide poisoning deaths. South Korea has the highest suicide rate (33.3 out of 100,000 population, based on 2011 data) among all OECD (Organization for Economic Co-operation and Development) countries and this suicide rate has been sharply increasing [13]. Based on South Korea’s country profile from the recent Global Burden of Disease Study 2010 [14], suicide is now the second most important cause of premature deaths as measured by years of life lost in South Korea. Suicide through pesticides is the second-most frequently used method, which accounted for 20.8% of total suicides, followed by hanging (50.9%) in South Korea during the study period of 2006 through 2010. South Korea has a relatively large proportion of suicide cases from pesticide poisoning compared with other developed countries such as Japan and Taiwan [15], [16].

The high rate of pesticide ingestion in suicide in South Korea may be explained by their easy accessibility in South Korea. Easy access to pesticides is believed make pesticide self-poisoning a preferred means of self-harm [17], [18]. Although South Korea does maintain regulations covering the buying or selling of restricted pesticides, in the absence of licensing requirements individuals experience little difficulty in bypassing the regulations and purchasing pesticides. The regional and seasonal variations in pesticide poisoning presented in this study may also corroborate this explanation of accessibility to pesticides. Considering that widespread access to pesticides may easily convert a number of impulsive acts into suicide by means of pesticide ingestion, restrictions on pesticides should be a priority for suicide prevention efforts in South Korea.

The study results showed that pesticide self-poisoning is a largely rural phenomenon and is the most common method of self-harm resulting in death, accounting for 47.4% of total rural suicides in South Korea. Rural suicides involving pesticides have been documented in other Asian countries such as Sri Lanka [19], Taiwan [20], and China [21]. Numerous aspects of rural life, such as socioeconomic disadvantages, limited availability of and access to emergency medical services, an aging population, and geographic and interpersonal isolation may all contribute to the elevated rates of suicide in rural areas [22], [23]. Such explanations may also be applied in a Korean context, compounded by easy access to pesticides. The number of people aged 65 years and older has been rapidly increasing in South Korea and the proportion of the elderly was 33.7% in rural areas in 2011 compared to the national average of 11.4% [24]. The elderly are the most vulnerable group in terms of self-destructive behavior and disproportionately represent the socially disadvantaged in contemporary Korean society [25]. The elderly make more serious suicide attempts using more-lethal methods [26] and access to lethal means such as pesticide ingestion seems to play an important role in suicides by the elderly in rural settings.

The results of the study revealed that men in rural areas were 2.4 times (16.58 vs. 6.85 per 100,000 population) more likely to die as a result of intentional pesticide poisoning than were women in rural areas. Corresponding figures for cities and metropolises were 2.6 times (8.04 vs. 3.09 per 100,000 population) and 2.8 times (3.05 vs. 1.09 per 100,000 population), respectively. The gender ratio in deaths by suicide stands at around 2.4 to 1 in South Korea [25] while the suicide rates in many high-income countries are typically more than three times higher among men than women [27]. Men have traditionally been more commonly engaged in occupations involving pesticides and have greater access to pesticides than do women. Thus, it can be suggested that the access to pesticides has allowed a relatively lower gender ratio in intentional pesticide poisoning in rural areas than that found in urban areas, as well as high suicide rates due to pesticide poisoning both in rural areas and across the country. In addition, this accessibility might have in part contributed to the higher suicide mortality rates and relatively lower gender ratio in suicide mortality compared with Western high-income countries. Moreover, our results that the rate of pesticide self-poisoning was the highest among men aged above 80 in rural areas (85.5 per 100,000 population) reflects a combined effect of risk factors for pesticide-related suicide among rural elderly men.

The poor quality of medical service in rural areas can be an important risk factor for elevated overall mortality, including mortality due to pesticide poisoning. One recent study reported that in South Korea rural areas showed higher mortality rates from major diseases, including poisoning, than did urban areas, although the prevalence of the diseases were similar in both rural and urban areas across the country [28]. Our results that the rural-metropolis ratio in the rate of pesticide poisoning appears to be more pronounced in mortality than in incidence support these results.

The large proportion of farmers in rural areas may also contribute to the high mortality and incidence of pesticide poisoning in such areas. In South Korea, an elevated occupational pesticide poisoning incidence of 24.7 per 100 population among male agricultural workers has been reported using nationwide survey data [29], and the incidence was significantly related with work-related factors such as poor personal hygiene practices and failure to make use of personal protective equipment [30]. Average pesticide application by unit of agricultural land in South Korea is also much higher than in other developed countries [31]. Furthermore, it has been suggested that the exposure to high levels of pesticides, including poisoning, experienced by agriculture workers and rural residents may result in an elevated risk of neuropsychiatric sequelae such as mood disorders, depression, and suicide attempts [32], [33]. Recently, we found that depressive symptoms [34] and suicidal ideation [35] were significantly associated with a history of acute occupational pesticide poisoning and that this relationship was further associated with the severity of the symptoms of poisoning among Korean male farmers.

We also found that highly toxic herbicides as a suicide method were more commonly used in rural areas than in metropolises, which may well explain higher ratio in mortality due to pesticide poisoning in rural areas. Paraquat is a non-selective herbicide that has been ranked as one of the most commonly used pesticides, and has been widely used as the main causative agent for suicide attempts in South Korea [36]. A survey based on nationwide 38 hospitals reported that the fatality of paraquat was 78%, whereas the average fatality of pesticides poisoning was as 22% in South Korea [37]. Although South Korea implemented the Act on Paraquat Regulations in 1999 and revised it in 2005, mortality due to paraquat was still seen to be high thereafter. Recently, the South Korean government banned the selling of paraquat from the end of 2012 but the paraquat sold prior to the ban continues to exist in South Korea due to the lack of further progressive policies such as recalling paraquat from the market and from farmers. Although paraquat poisoning cases can be anticipated to decrease with the new government policy, it is important to monitor the change in suicide by pesticide ingestion for the identification of alternative pesticides in the future.

This study features an important strength in terms of the simultaneous report of nationally representative figures on mortality from and the incidence of pesticide poisoning. However, because the two databases used in this study were not individually linked, certain limitations exist in the identification of the fatality of pesticide poisoning or related risk factors. In addition, the National Health Insurance claims data may not include deaths occurring outside hospitals. Patients who had been referred to other hospitals or voluntarily discharged from hospitals in the National Health Insurance claims data were not determined the status of death in our analysis.

Another limitation of our study is that the death certificates and National Health Insurance data we used were mainly for administrative purposes, therefore, they do not include detailed information for research such as the detailed place of death, time to death from ingestions and other co-morbid factors. In particular, the National Health Insurance claims data had no information regarding intent of poisoning and suicide, which hinders further analyses. In addition, the magnitude of pesticide poisoning based on hospital records or mortality data in this study may underestimate the full impact of pesticide poisoning.

Suboptimal levels of validity for the coding of pesticide poisoning cases may also be a potential weakness of our study. However, in South Korea, the death registration rate has reached near completeness and 97.5% of all deaths in the national registered death data in 2011 are confirmed by a physician’s diagnosis [38]. According to a validation study on the diagnostic codes found in the National Health Insurance claims database in South Korea for the years 1999 and 2001, the consistency of diagnostic codes with medical records was determined to be approximately 70% and tended to be higher for hospital admissions and severe conditions than for outpatients or mild cases [39].

## Conclusions

We have demonstrated through the national death and healthcare utilization data that the magnitude of acute pesticide poisoning in South Korea is higher than that in other developed countries. The majority of pesticide poisoning deaths were the result of intentional poisoning; in particular, elderly suicide by pesticide ingestion in rural areas was shown to be a serious social problem. Easy access to pesticides and the lack of management of suicide by pesticide ingestion in rural area are suggested as major factors related with the high rate of pesticide poisoning in South Korea. Therefore, intensive intervention efforts, such as the strict regulation of toxic pesticides and prevention efforts directed at controlling suicide are critically needed to reduce the burden of pesticide poisoning in South Korea.

## Supporting Information

Appendix S1 Contains the files: Table S1. Number of population and age-specific death rate for pesticide poisoning in 2010. Table S2. Age-standardized death rates of pesticide poisoning in 2010 by WHO World Standard Population.
(DOCX)
