# Supplementary material for: Mortality from and Incidence of Pesticide Poisoning in South Korea: Findings from National Death and Health Utilization Data between 2006 and 2010
Source: PLoS One. 2014 Apr 17;9(4):e95299. doi: 10.1371/journal.pone.0095299 (PMC3990630; doi:10.1371/journal.pone.0095299)
Supplement: Appendix S1 — Contains the files: Table S1. Number of population and age-specific death rate for pesticide poisoning in 2010. Table S2. Age-standardized death rates of pesticide poisoning in 2010 by WHO World Standard Population. (DOCX) [file pone.0095299.s001.docx]

**Appendix**

**Calculation of age-adjusted rate for pesticide poisoning**

We used population data by gender, 10-year age groups, and the 251 administrative districts obtained from Statistics Korea (Statistics Korea, http://kostat.go.kr). This data is based on population of each year in South Korea. Mortality and incidence rates were calculated using the population data as denominators and deaths from and patients of pesticide poisoning as numerators and the rates were directly standardized to 10-year age groups using the 2000 World Standard Population (Ahmad et al., 2001. WHO GPE Discussion Paper series: No. 31. Age standardization of rates: a new WHO Standard). Appendix Table S1 shows the crude death rate of pesticide poisoning using population data at the midpoint of 2010 in South Korea. Appendix Table S2 shows the process of calculation of age-standardized death rates for 2010 by the direct method. Age-standardized rate per 100,000 population was calculated as total number of expected deaths.

Appendix Table S1. Number of population and age-specific death rate for pesticide poisoning in 2010

| Age group | Population^a^ | Pesticide poisoning deaths | Pesticide poisoning rates/100,000 (A) |
| --- | --- | --- | --- |
| 0-19 | 11,584,399 | 5 | 0.04 |
| 20-29 | 6,866,956 | 32 | 0.47 |
| 30-39 | 8,370,549 | 169 | 2.02 |
| 40-49 | 8,844,352 | 380 | 4.30 |
| 50-59 | 7,066,823 | 469 | 6.64 |
| 60-69 | 4,191,329 | 681 | 16.25 |
| 70-79 | 2,618,525 | 951 | 36.32 |
| 80+ | 972,733 | 519 | 53.35 |
| Total | 50,515,666 | 3,206 | 6.35 |

^a^Population of 2010 in South Korea (Statistics Korea, http://kostat.go.kr)

Appendix Table S2. Age-standardized death rates of pesticide poisoning in 2010 by WHO World Standard Population^a^

| Age group | Pesticide poisoning rates/100,000 (A) | World Standard Population (B)^a^ | Expected deaths/100,000 (AⅹB) |
| --- | --- | --- | --- |
| 0-19 | 0.04 | 0.3462 | 0.01 |
| 20-29 | 0.47 | 0.1615 | 0.08 |
| 30-39 | 2.02 | 0.1476 | 0.30 |
| 40-49 | 4.30 | 0.1263 | 0.54 |
| 50-59 | 6.64 | 0.0992 | 0.66 |
| 60-69 | 16.25 | 0.0668 | 1.09 |
| 70-79 | 36.32 | 0.0373 | 1.35 |
| 80+ | 53.35 | 0.0154 | 0.82 |
| Total |  |  | 4.85^b^ |

^a^The 2000 World Standard Population (Ahmad et al., 2001)

^b^The standardized death rate for pesticide poisoning per 100,000
